# A Piezoelectric Tactile Sensor for Tissue Stiffness Detection with Arbitrary Contact Angle

**DOI:** 10.3390/s20226607

**Published:** 2020-11-18

**Authors:** Yingxuan Zhang, Feng Ju, Xiaoyong Wei, Dan Wang, Yaoyao Wang

**Affiliations:** College of Mechanical and Electrical Engineering, Nanjing University of Aeronautics and Astronautics, Nanjing 210016, China; zhangyingxuan@nuaa.edu.cn (Y.Z.); weixiaoyong@nuaa.edu.cn (X.W.); wangdan_053@nuaa.edu.cn (D.W.); yywang_cmee@nuaa.edu.cn (Y.W.)

**Keywords:** tactile sensor, piezoelectric sensor, stiffness detection, arbitrary contact angle

## Abstract

In this paper, a piezoelectric tactile sensor for detecting tissue stiffness in robot-assisted minimally invasive surgery (RMIS) is proposed. It can detect the stiffness not only when the probe is normal to the tissue surface, but also when there is a contact angle between the probe and normal direction. It solves the problem that existing sensors can only detect in the normal direction to ensure accuracy when the degree of freedom (DOF) of surgical instruments is limited. The proposed senor can distinguish samples with different stiffness and recognize lump from normal tissue effectively when the contact angle varies within [0°, 45°]. These are achieved by establishing a new detection model and sensor optimization. It deduces the influence of contact angle on stiffness detection by sensor parameters design and optimization. The detection performance of the sensor is confirmed by simulation and experiment. Five samples with different stiffness (including lump and normal samples with close stiffness) are used. Through blind recognition test in simulation, the recognition rate is 100% when the contact angle is randomly selected within 30°, 94.1% within 45°, which is 38.7% higher than the unoptimized sensor. Through blind classification test and automatic k-means clustering in experiment, the correct rate is 92% when the contact angle is randomly selected within 45°. We can get the proposed sensor can easily recognize samples with different stiffness with high accuracy which has broad application prospects in the medical field.

## 1. Introduction

With the development of medical treatment and the advancement of robot technology, robot-assisted minimally invasive interventional surgery (RMIS) is becoming popular. It has the advantages of small trauma, quick recovery and high accuracy. Compared with traditional surgery, doctors cannot touch tissue directly in RMIS, which may cause misdiagnosis or wrong resection. Since the lumps are often harder than the surrounding normal tissues, doctors can usually directly distinguish the lump by the difference in stiffness felt. [1,2] In order to distinguish different stiffness in RMIS, a tactile sensor for tissue stiffness detection is essential. 

There are many kinds of tactile sensors that can be used to detect force and stiffness. C. H. Chuang [3] designs a minimized tactile sensor which can be mounted on an endoscope to detect tissue with abnormal stiffness. A special piezoelectric system mounted on conventional biopsy needles proposed in [4] is designed to evaluate abnormal tissues when penetrating tissue. A force sensor proposed in [5,6] is used to measure instrument interaction forces in a beating heart. A unique structure of the Fiber Bragg Grating (FBG) force sensor proposed by Lv [7], composed with a central optical fiber, can detect axial force with high resolution and small error, but the structure will deform when detecting, which will easily lead to fatigue damage of the probe. The FBG three-dimensional force sensor for palpation proposed by Li [8] consists of five FBGs arranged symmetrically. Since the change of the center wavelength of the FBG is proportional to the strain applied to the optical fiber, the lateral force and longitudinal force applied to the probe can be measured by the change in wavelength caused by the stretching or compression of a pair of symmetrical fibers, and the axial force can be measured by the changes of five fibers. However, due to the complex structure, it is difficult to integrate into the end of the flexible surgical robot arm. In [9], the tactile sensor detects tumors based on strain gauges, which is composed of four strain gauges with different Young’s modulus. The contact force can be obtained by measuring the resistance shift. The vision-based detection method in [10] uses tissue deformation with supervised learning providing the surgeon with feedback. However, they all need enough force or deformation to press the tissue for detection, which may cause additional harm. The piezoelectric sensor, based on resonant frequency shift in [11,12] which can detect only by contacting tissue, is safer because it avoids the possibility of tissue damage and large deformation. Additionally, it has simple structure, which can be self-driven and self-sensing. However, the resonant frequency is high [12], which may increase the influence of equivalent mass and result in inaccuracy. What is more, none of them explain the condition when detected with a contact angle. 

So far, most tactile sensors in literature can detect the stiffness of tissue only when the contact is in the normal direction. However, most MIS procedures are performed in a confined space such as the transurethral resection of bladder tumor (TURBt) and uterine fibroid embolization (UFE). The limited degree of freedom (DOF) of the instrument causes the tactile sensor to become unable to be placed in the normal direction to contact the tissue surface, which may lead to lump undetectable. The method of achieving stiffness detection and lump identification under multiple constraints is particularly important. However, the use of existing tactile sensors is mostly limited by the contact angle between the probe and normal direction of tissue surface. The stiffness palpation method proposed in [13] can distinguish vein from surrounding tissue. Additionally, method in [14] can detect the shape of the measured tissue. However, they can only limit the sensor probe in the normal direction of the tissue surface to ensure accuracy of stiffness detection. The sensor proposed in [15] can detect force from different directions, but the detectable angle is small, and the contact force is not small enough. For sensors that only need to contact the tissue to detect stiffness, there is little related work discussing how the contact angle affects stiffness detection, and it is not proved how to reduce the deviation caused by the angle. 

This paper proposes a piezoelectric tactile sensor that can not only detect the stiffness when the probe is normal to the tissue surface, but also when there is a contact angle relative to the normal direction. The detection model with a contact angle is proposed in Section 2. In order to reduce the influence of the angle on stiffness detection, the sensor parameters are designed and optimized. Additionally, sensor detection performance is verified in simulation and experiments in Section 3 and Section 4.

## 2. Materials and Methods

A piezoelectric sensor is preliminarily designed, and the sensing principle explains how the sensor works. In order to know the relationship between stiffness and contact angle *α*, a stiffness detection method is proposed. Based on that, parameters of the sensor are designed and optimized to minimalize the influence of angle change on detection.

### 2.1. Sensor Structure

**Figure 1:**
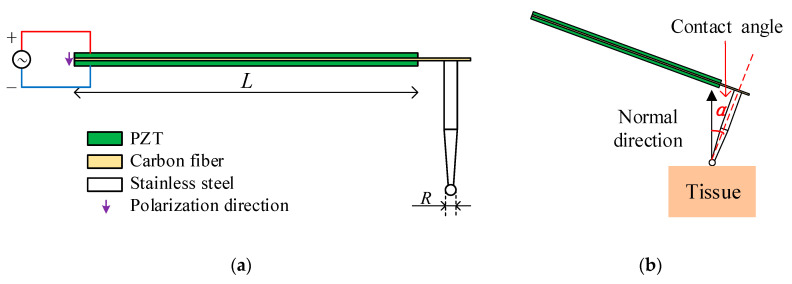
(**a**) Structure of piezoelectric tactile sensor. (**b**) Detect tissue with contact angle *α*, which refers to the angle between sensor probe and the normal direction of tissue surface.

This paper proposes a piezoelectric bimorph tactile sensor, and the structure is shown in Figure 1a. The lead zirconate titanate piezoelectric ceramics (PZT) is used as a sensitive element due to its superior piezoelectric properties. Other materials such as potassium sodium metaniobate, barium strontium metaniobate, Ag PDA and Ag ZnO [16] are not mature enough to be used for sensing. From the current applicable research results, their performance is still not as good as PZT. PZT plays the role of driving and sensing at the same time. Its length is L. The middle of the bimorph is a carbon fiber interlayer. The tip of the stainless-steel probe is a ball that contacts with the tested tissue, and its radius is R. The contact angle *α*, as shown in Figure 1b, refers to the angle between sensor probe and the normal direction of tissue surface.

### 2.2. Sensing Principle and Detection Method

#### 2.2.1. Sensing Principle

**Figure 2:**
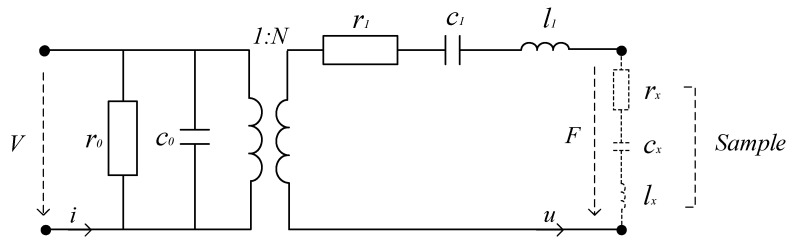
Equivalent circuit of sensing system.

Due to the inverse piezoelectric effect, applying an AC voltage in the polarization direction of the PZT (as shown in Figure 1) will drive the sensor to vibrate. When the vibrating sensor contacts the tested tissue, the sensor and the tested tissue form a new system. Compared with the system before contacting, mechanical impedance has changed. Through the positive piezoelectric effect, changes in mechanical impedance are transformed into changes in electrical impedance. The change of impedance directly causes the shift of the system’s resonance frequency which is usually related to the stiffness of the tested tissue. A simplified equivalent circuit of sensing system in resonance mode is shown in Figure 2 [17].

The electromechanical coupling is represented as an ideal transformer, the primary side represents the pure dielectric characteristics of the system, and the secondary side reflects the mechanical characteristics. In the secondary side, the viscous loss is equivalent to the resistance *r*_1_, the damping is equivalent to the capacitance *c*_1_, and the vibration mass is equivalent to the inductance *l*_1_.

When the sensor system contacts the tissue, the part of the tissue contacted can be equivalent to resistance *r_x_*, capacitance *c_x_* and inductance *l_x_*. The resonance frequency of the sensor is determined by *r*_1_, *c*_1_, and *l*_1_ before contacting, and changes to a new value after the equivalent impedance *Z_x_* of the tissue is loaded into the circuit. The impedance *Z_x_*, resistance *r_x_*, capacitance *c_x_* and inductance *l_x_* can be expressed as [17]:

(1) $$\begin{array}{l} Z_{x}=r_{x}+j(\omega l_{x}-\frac{1}{\omega c_{x}})\\ r_{x}=\frac{2\sqrt{2}a_{12}}{\pi (1-v_{x})\sqrt{1+v_{x}}}\sqrt{\rho_{x}E_{x}}S\\ \frac{1}{c_{x}}=\frac{2}{\sqrt{\pi }(1-v_{x}{}^{2})}E_{x}\sqrt{S}\\ l_{x}=\frac{4a_{11}}{\pi^{1.5}(1-v_{x})}\rho_{x}S^{1.5} \end{array}$$

where *ω* is the angular frequency, *v_x_* is the Poisson’s ratio, *a*_11_ and *a*_12_ are coefficients related to Poisson’s ratio, *E_x_* is the Young’s modulus of the tested tissue, and *S* is the area of the contact part between the sensor probe and the tissue.

After the sensor touches the tested tissue, the impedance of the system changes. The resonance frequency of the system shifts due to the change which can be measured.

The frequency shift after the sensor touches the tissue can be expressed as [18,19]:

(2) $$\Delta f=-\frac{C(\omega l_{x}-\frac{1}{\omega c_{x}})}{2\pi LZ_{1}}$$

Substituting Equation (1) into (2) finally obtains the relationship between the frequency shift Δ*f* and the characteristics of the tested tissue:

(3) $$\Delta f=\frac{CE_{x}\sqrt{S}}{\pi^{1.5}\omega LZ_{1}(1-v_{x}{}^{2})}$$

#### 2.2.2. A New Detection Method and Its Optimization

**Figure 3:**
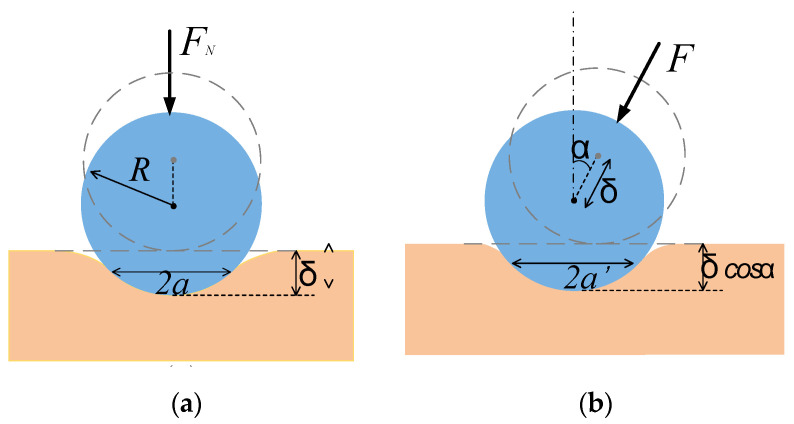
The contact model between the sensor probe and the tissue surface (**a**) in normal direction and (**b**) angle *α*.

The contact between the probe of the sensor and the tested tissue can be expressed as static contact and dynamic contact. Static contact means that a small force makes the probe contact the tissue at a certain indentation depth; dynamic contact means that an AC voltage is applied to the sensor which causes a small vibration between probe and tissue. However, for measuring the stiffness of object with a large anisotropy factor and Poisson’s ratio, the contact between the sensor and tissue can be approximated by static contact [20]. Biological tissue is anisotropic and viscoelastic. The anisotropy factor and Poisson’s ratio on the surface of most tissues is relatively large, generally ranging from 0.7 to 0.99 [21], and Poisson’s ratio is generally around 0.5. Therefore, the normal contact between the probe and the tissue can be simplified to the model shown in Figure 3a [17].

As shown in Figure 3a, under the normal force *F_N_*, the sensor has a normal relative displacement Δ to the surface of the tissue, which is called indentation depth. Since the probe is spherical with a radius of *R*, the contour of the contact part can be approximated as a circle with a diameter of 2*a*, *a* *≤ R*. From the geometric relationship of contact, we can get:

(4) $$\begin{array}{l} \delta =2R-2\sqrt{R^{2}-a^{2}}\\ a=\sqrt{R\delta -0.25\delta^{2}} \end{array}$$

When the sensor’s DOF is limited, it cannot detect tissue in the normal direction but at angle *α*, the probe produces a displacement of Δ in this direction. The contour of the contact part can still be approximately circular, with a diameter of 2*a’*, as shown in Figure 3b. The contact radius and contact area are:

(5) $$a^{\prime }=\sqrt{R(\delta \cos\alpha )-0.25{\left(\delta \cos\alpha \right)}^{2}}$$

(6) $$S=\pi [R(\delta \cos\alpha )-0.25{\left(\delta \cos\alpha \right)}^{2}]$$

It can be seen from Equation (6) that if the depth Δ is maintained at a constant value, the larger the angle *α* (*α* < 90°), the smaller the contact area S.

Substituting Equation (6) into (3):

(7) $$\Delta f=\frac{CE_{x}\sqrt{R(\delta \cos\alpha )-0.25{\left(\delta \cos\alpha \right)}^{2}}}{\pi \omega LZ_{1}(1-v_{x}{}^{2})}$$

It can be seen from the Equation (7) that the stiffness of the tested tissue is proportional to the frequency shift. When the sensor presses the tissue at a contact angle *α*, the larger the angle *α*, the smaller the frequency shift Δ*f*. However, the change of Δ*f* can be reduced.

**Figure 4:**
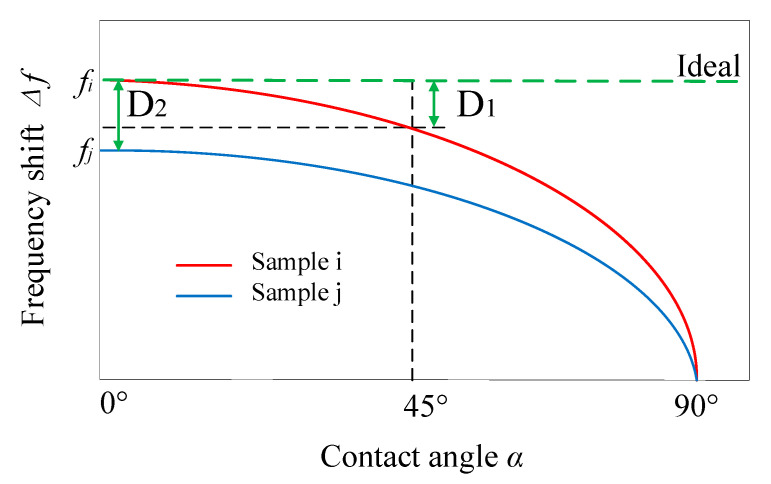
The schematic image of Δ*f-α* function.

The sensor’s Δ*f* is not only related to the stiffness and the angle during detection, but also related to the parameters of the sensor itself: the length *L* of PZT and the radius *R* of the probe. Through the design and optimization of these two items, the influence of *α* on Δ*f* can be reduced. In order to prevent the slip phenomenon, we only discuss the angle in the range of *α* ∈ [0°, 45°]. Taking the angle *α* in Equation (7) as the independent variable, the schematic image of Δ*f-α* function is shown in Figure 4.

To optimize the performance of the sensor, two goals of sensor parameters optimization are: (1) As shown in Figure 4, the difference between Δ*f*_0°_and Δ*f*_45°_ is close to 0 (*D*_1_ is close to 0), to ensure that the angle change has the smallest impact on the detection result. (2) When tissues with different stiffness are tested, the difference between Δ*f_i_* and Δ*f_j_* should be larger than *D*_1_ to ensure that they can be reliably distinguished, as shown in *D*_2_ in Figure 4. According to these goals, the corresponding expression can be written:

(8) $$D_{1}=\Delta f(0{}^{\circ})-\Delta f(45{}^{\circ})=\frac{CE_{x}(\sqrt{R\delta -0.25\delta^{2}}-\sqrt{0.7R\delta -0.125\delta^{2}})}{\pi \omega lZ_{1}(1-v_{x}{}^{2})}$$

(9) $$\frac{D_{1}}{D_{2}}=\frac{\Delta f_{i}(0{}^{\circ})-\Delta f_{i}(45{}^{\circ})}{\Delta f_{i}-\Delta f_{j}}=\frac{E_{x(i)}(\sqrt{R\delta -0.25\delta^{2}}-\sqrt{0.7R\delta -0.125\delta^{2}})}{(E_{x(i)}-E_{x(j)})\sqrt{R(\delta \cos\alpha )-0.25{\left(\delta \cos\alpha \right)}^{2}}}<1$$

where *i* and *j* represent samples with different stiffness. Stiffness of sample *i* is larger than sample *j*.

Solve Equation (8) *D*_1_ ≥ 0, get *R* ≥ 0.42Δ. In Equation (9), select the extreme case (large angle and close stiffness value) for discussion, set *α* = 0°, *E_x(i)_* = 1.2*E_x(j)_*, the solution is *R < 2.5*Δ. In order to ensure the contact surface is approximately circular, control *R* *≥* Δ. Therefore, take *R =* Δ. For the *L*, two sets of parameters are selected for comparison in simulation.

## 3. Results

### 3.1. Simulation Studies

In order to test the sensor performance on distinguishing different stiffness samples with arbitrary contact angle, effect of optimization and blind recognition ability, simulation is designed.

#### 3.1.1. Test of Distinguishing Samples with Different Stiffness

A model of the sensor and the tested samples was established, and the model was simulated in Ansys Workbench. Sensor structure is shown in Figure 1. The PZT length *L* = 35 mm is preliminarily chosen, and probe tip radius and indentation depth *R =* Δ = 0.5 mm are selected. The materials of sensor are shown in Table 1.

Five samples with different stiffness are set, including the normal tissue with the smallest stiffness (sample 1) and lumps (samples 2, 3, 4 and 5) [22], and their stiffness is shown in Table 2. The stiffness of normal tissue is set between fat and muscle, and the stiffness of lumps is set 5~15 times that of normal tissue.

**Figure 5:**
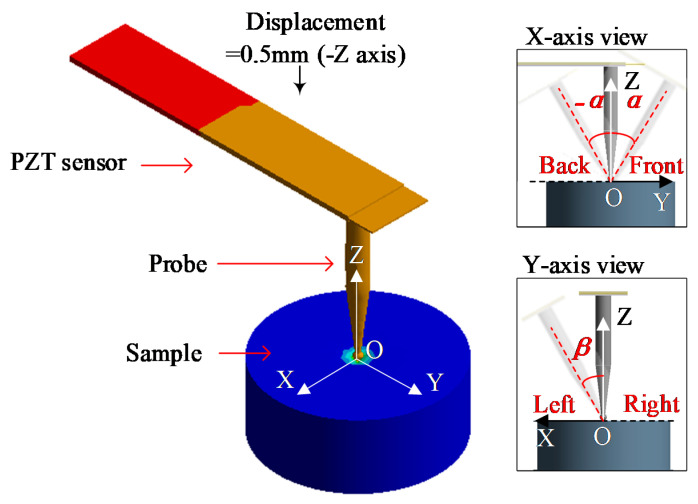
Simulation of detection in the front, back, and left directions with contact angle.

The contact angle *α* is set to 0°, 15°, 30° and 45°, respectively. The contact with the sample is set as frictional contact, and the friction coefficient is 0.1 [23]. In order to simulate the various contact situations in the actual surgery, the sensor probe and the sample have contact angles *α* (-*α* or *β*) in the front, back, and left (symmetrical with right) directions, as shown in Figure 5.

Apply +5 V AC sweep voltage to the upper and lower sides of the PZT which means that the vibration amplitude is much smaller than the indentation depth. Set the sweep range in the harmonic response analysis to be near the system resonance frequency. Because the system impedance will change suddenly at the resonance frequency, the frequency-impedance curve is used to record the resonance frequency, and then subtract with the no-load (before contacting the sample) resonance frequency to obtain the frequency shift Δ*f* under different conditions. The no-load resonance frequency is 140.4 Hz in simulation.

**Figure 6:**
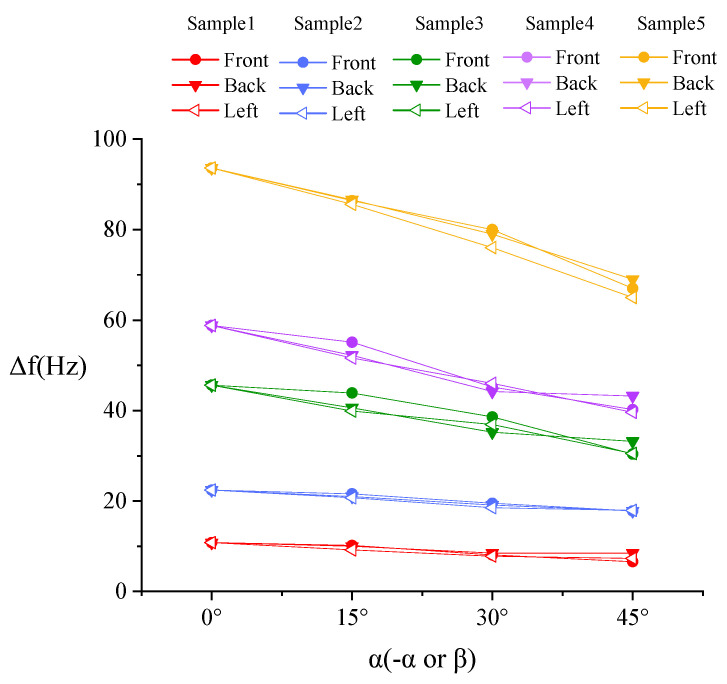
Simulation result of frequency shift Δ*f*-contact angle *α* (-*α* or *β*) in three different directions.

**Figure 7:**
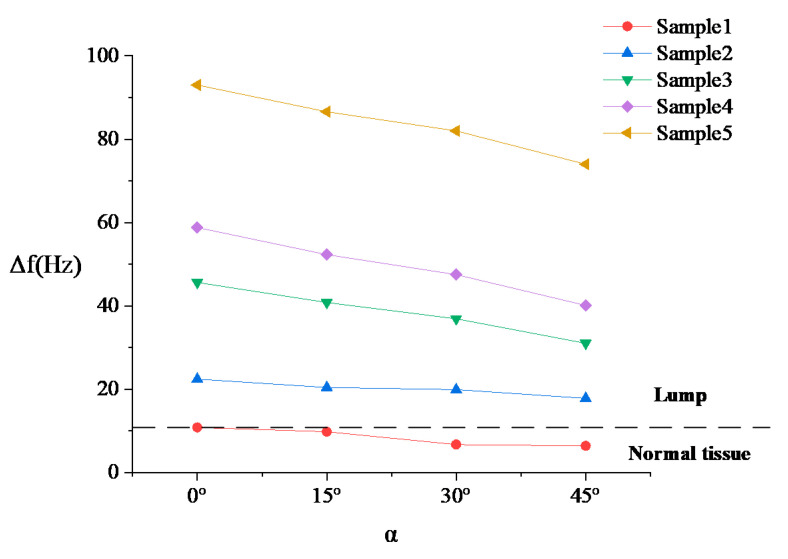
Simulation result of distinguishing different stiffness samples with different contact angles.

Figure 6 shows that the detected direction has no significant influence on the result, so the resonance frequency results measured in the three directions are averaged in Figure 7 (the angle is represented by *α*) for facilitate analysis.

It can be seen from Figure 7 that the relationship of Δ*f-α* is consistent with the Equation (7). Obviously, samples with different stiffness can be distinguished and lump samples can be easily distinguished from normal tissue sample.

Sensor sensitivity refers to the ratio between the resonant frequency shift and the change in stiffness of the tissue, which determines the effective detection range of the sensor. The sensitivity of the proposed tactile sensor is 134.11 HZ/MPa in the range from 0.045 Mpa to 0.649 Mpa.

#### 3.1.2. Effect of Optimization

**Figure 8:**
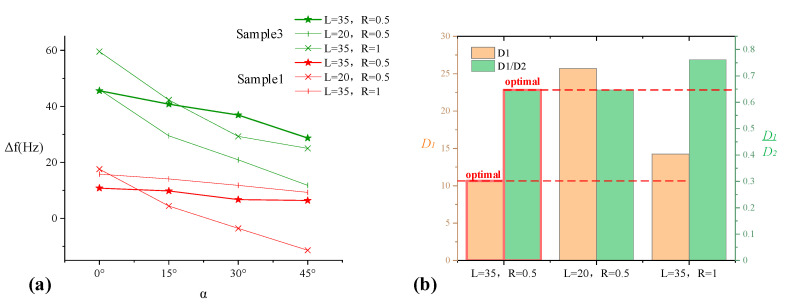
(**a**) Comparison of Δ*f-α* function of two samples with different parameters and (**b**) Optimal amount comparison.

In order to verify the optimization effect and further optimize the length *L*, another two sensors with different parameters are taken for comparison. The indentation depths all choose Δ = 0.5 mm. The first group selects parameters PZT length *L* = 35 mm and the probe radius *R* = 0.5 mm, the second group selects *L* = 20 mm and *R* = 0.5 mm, the third group selects *L* = 35 mm and *R* = 1 mm. Select normal tissue sample 1 and lump sample 3 for simulation, and the process is the same as above. The comparison of three sets of parameters is shown in Figure 8.

From the Δ*f-α* comparison in Figure 8a, the frequency shift of the sensor with *L* = 35 mm and *R* = 0.5 mm is minimally affected by the angle change, and the two samples can be distinguished clearly. Comparison of optimization parameters *D*_1_ and *D*_1_*/D*_2_ is shown in Figure 8b, the sensor with *L* = 35 mm and *R* = 0.5 mm has the optimal parameters.

#### 3.1.3. Sample Blind Recognition Test in Arbitrary Angle

**Figure 9:**
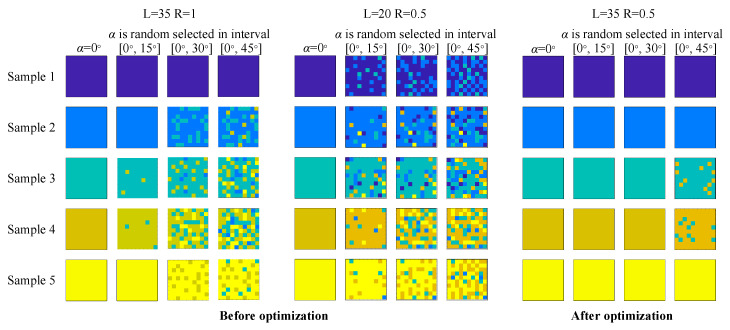
Sample surface points recognition result in simulation before sensor optimization and after optimization.

In order to prove the ability of the optimized sensor to recognize samples with different stiffness when the contact angle is from 0° to 45°, five samples (the same as mentioned before) are used as the tested objects, comparing the blind recognition effect of the optimized sensor (*L* = 35, *R* = 0.5) and the unoptimized sensors (*L* = 35, *R* = 1 and *L* = 20, *R* = 0.5). The size of each sample is set to 10 × 10 cm, and the surface is discretized into a 10*10 lattice, and the stiffness (expressed in Δ*f*) of each point is detected one by one. Take the contact angle as 0° (surface normal direction), and random contact angle in the interval [0°, 15°], [0°, 30°] and [0°, 45°] to detect the stiffness value of each point of each sample. The stiffness value of each sample at the boundary of the interval is used as the recognition range of the sample surface point. That is, when sample 1 is tested, if the stiffness value of a certain point is within the recognition range of sample 1, this point will be judged as sample 1. If it is also in the range of sample 2 at the same time, it may be misjudged as sample 2. The boundaries of the stiffness range of the five samples are obtained by simulation. The results of normal detection and random angle detection are shown in Figure 9.

Figure 9 shows that as the interval of the random angle increases, the recognition rate of unoptimized sensors gradually decreases. Two sensors before optimization show that their recognition rate is 100% in normal, 96.7% in [0°, 15°],79.6% in [0°, 30°] and 73.7% in [0°, 45°] for *L* = 35 *R* = 1 sensor, and 100% in normal, 77.3% in [0°, 15°], 67.0% in [0°, 30°] and 55.4% in [0°, 45°] for *L* = 20 *R* = 0.5 sensor. The sample cannot be effectively recognized when there is an arbitrary contact angle. The sample recognition rate of the optimized sensor remains stable around 100% (normal: 100%, [0°, 15°]: 100%, [0°, 30°]: 100%, [0°, 45°]: 94.1%), far better than the recognition rate before optimization.

### 3.2. Experiment Studies

Based on simulation, an experiment is designed, a piezoelectric sensor is fabricated. Five soft silica gel samples with different stiffness are made. The stiffness of samples consistent with the simulation. The actual stiffness measured by the 00-type hardness tester is shown in Table 3.

**Figure 10:**
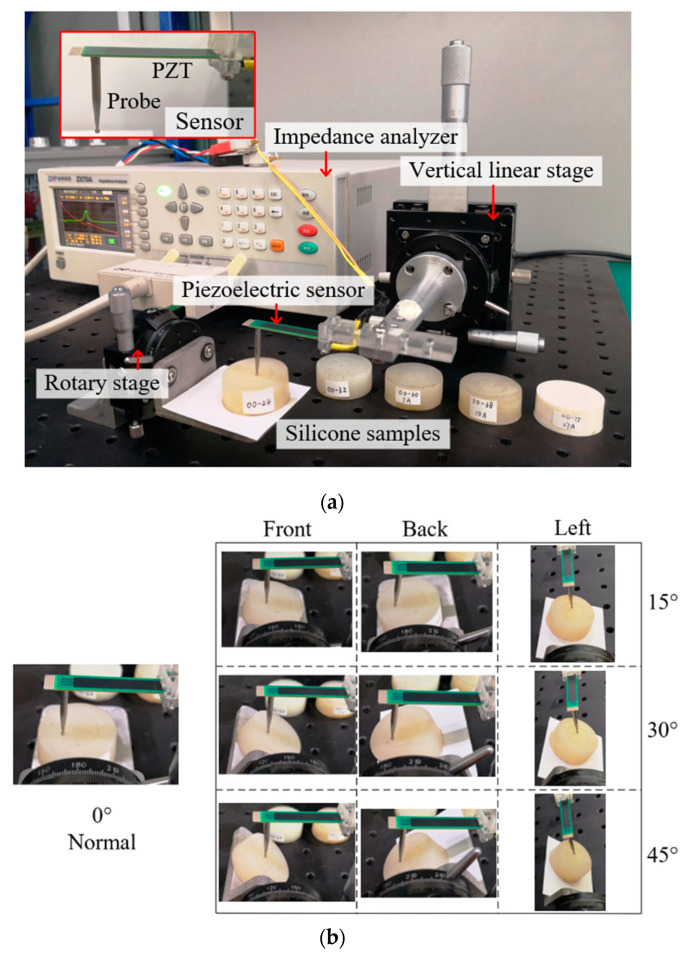
(**a**) Experiment settings. (**b**) Sensor in different directions and different angles.

As shown in Figure 10a, the vertical linear stage is used to control the sensor to move up and down to leave or contact the sample, and each test ensures that the indentation depth is 0.5 mm. The rotary stage is used to control the contact angle. As shown in Figure 10b, the contact angle of sample surface is controlled to be 0°, 15°, 30° and 45° in the three directions, and the sensor probe is vertical, so that the relative angle between the probe and the normal direction of surface is the set angle. The impedance analyzer is used to detect the resonance frequency of the sensing system.

**Figure 11:**
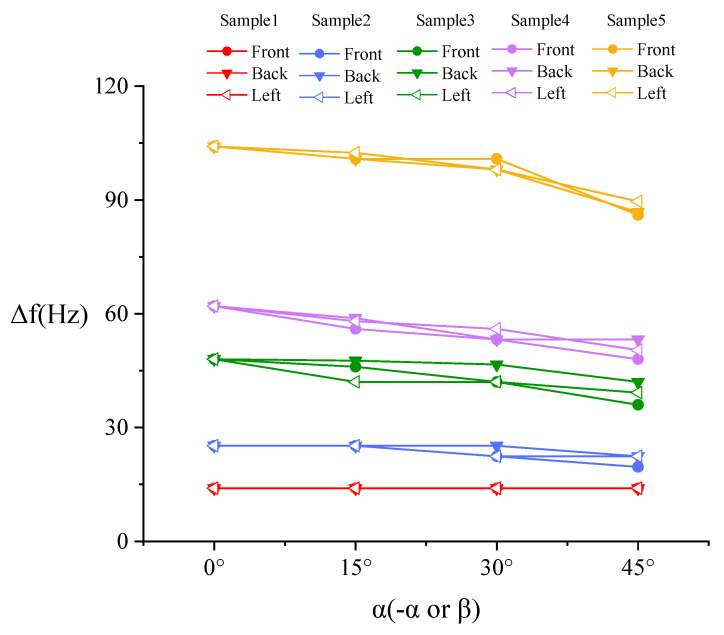
Experiment result of Δ*f-α* (*-α* or *β*) in three different directions.

**Figure 12:**
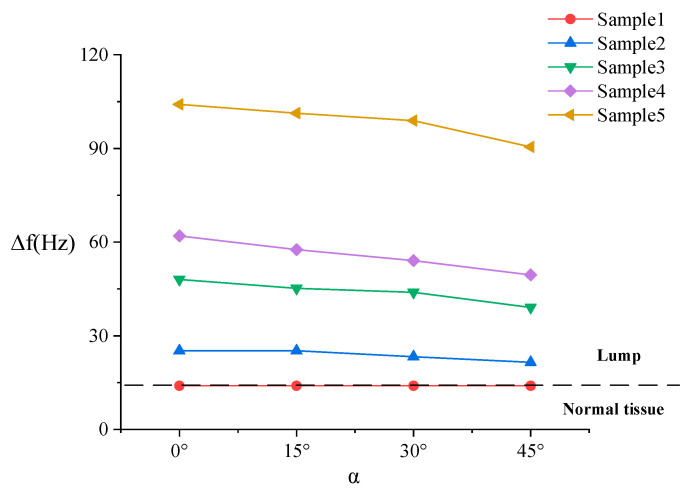
Experiment result of distinguishing different stiffness samples.

The resonance frequency of the un-load sensor is 137.6 Hz, which is approximately the same as the simulation result. Figure 11 shows that the detected direction has no significant influence on stiffness detection, so the resonance frequencies measured in the three directions are averaged in Figure 12 (the angle is represented by *α*) to facilitate analysis.

It can be seen from Figure 12 that the experimental results are consistent with the simulation results, and both are consistent with the theoretical curve. When the angle changes from 0° to 45°, the decrease in frequency shift is small. Additionally, the smaller the stiffness of the lump, the smaller the influence of the angle change on the stiffness result. Obviously, five samples can be distinguished even though the contact angle changes to 45°. Additionally, the lump samples and the normal tissue sample can be well distinguished.

**Figure 13:**
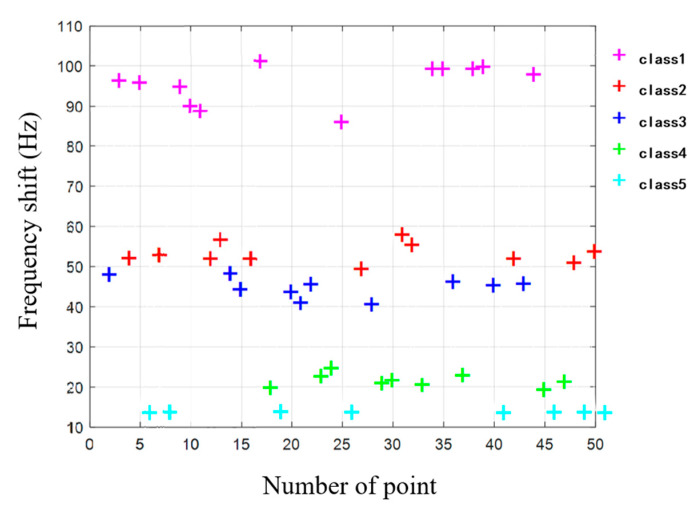
Classification result using k-means clustering algorithm for automatic samples recognition in experiment.

In order to prove the sensor’s ability to blindly recognize samples with different stiffness, the labels of the five samples are torn off and the order was disturbed. The proposed sensor is used to randomly detect a sample with a contact angle within 45°. Each random detection process includes randomly selecting a sample of unknown stiffness, randomly selecting a point on this sample (the sample stiffness is uniform), and testing the stiffness of this point at a random contact angle. After each test is completed, each sample is shuffled for the next test, repeating 50 times. These 50 stiffness test results (expressed in frequency shift) are imported into k-means clustering algorithm for automatic classification. The classification result is shown in Figure 13.

From Figure 13, it can be seen that 50 points are divided into five categories, corresponding to five samples, and four of them are misclassified. The correct rate is 92%.

## 4. Discussion

In this paper, a piezoelectric tactile sensor for detecting tissue stiffness in RMIS is proposed, whose detection principle is based on the resonant frequency change of the system after making contact with the tissue. The stiffness can be detected without causing damage to the tissue. The outstanding feature of the sensor is that it can detect the stiffness and distinguish different stiffness samples not only in the normal direction, but also when there is a contact angle between probe and normal direction of tissue surface, solving the problem that when the DOF of surgical instruments is limited, the sensor cannot detect in normal direction. For the traditional tactile sensor, when it deviates from the normal direction, the stiffness cannot be detected, or the accuracy is greatly reduced. The proposed sensor solves this problem well.

Through theoretical analysis, the principle of sensing and the stiffness detection method with a contact angle are obtained. The sensor system’s resonance frequency shift is proportional to the stiffness of the tested tissue and is negatively related to the contact angle. The parameters of the sensor are optimized to minimalize the influence of angle on stiffness. In order to test the optimized sensor performance, a simulation and an experiment are designed. Normal tissue sample and lump samples are set. When the angle changes from 0° to 45°, samples with different stiffness can be clearly distinguished. The sample recognition rate of the optimized sensor is 100% when the contact angle is selected randomly within 30° (including in the normal direction) in simulation, 94.1% within 45° (92% in experiment), which is 38.7% higher than the unoptimized sensor. It can be seen that the proposed sensor is hardly affected by angle when detecting stiffness. Additionally, it has abilities to distinguish different stiffness, distinguish lump from normal tissue, and recognize different stiffness samples with high precision. It signifies that the stiffness detection is no longer restricted by space and DOF, and that the sensor can detect tumors in any posture with high accuracy.

Although the sensor has been well verified in this study, there are still some areas that need to be improved. The proposed sensor is sensitive within the stiffness range of samples used in tests, but the sensitivity can be further analyzed with a larger stiffness range with continuous values. For the contact angle aspect, subsequent study will focus on effectively compensating the influence of the contact angle on the sensor’s detection to obtain more accurate stiffness results. For sensor products, an isolation coating will be used for insulation and protection. Additionally, to further test the performance of the sensor, the sensor will be attached to the end of the surgical robot and a porcine bladder will be used. At the same time, the sensor will be further studied how to be more compact to meet the needs of more minimally invasive surgery.

## 5. Conclusions

For the proposed piezoelectric sensor, we establish a new detection model with contact angle to optimize the sensor’s parameters. It makes the sensor insensitive to angle during detection, so as to meet the requirement of detecting stiffness in any posture. At the same time, the accuracy of stiffness identification is verified, proving that it has the potential to be used in interventional surgery with limited space.

## Figures and Tables

**Table 1:** Simulation material settings.

Part of Sensor	Material
Probe	Stainless steel
PZT	PZT-5A
Interlayer	Carbon fiber
Samples	Mooney-Rivlin

**Table 2:** Simulation sample settings.

Samples	Stiffness (MPa)
1	0.0450
2	0.104
3	0.235
4	0.320
5	0.649

**Table 3:** Experiment sample settings.

Samples	Stiffness (00)
1	00–24
2	00–32
3	00–60
4	00–68
5	00–75

## References

[1] Nagy T.D., Haidegger T. Recent Advances in Robot-Assisted Surgery: Soft Tissue Contact Identification. Proceedings of the 2019 IEEE 13th International Symposium on Applied Computational Intelligence and Informatics (SACI).

[2] Bandari N., Dargahi J., Packirisamy M. (2020). Tactile Sensors for Minimally Invasive Surgery: A Review of the State-of-the-Art, Applications, and Perspectives. IEEE Access.

[3] Chuang C.-H., Li T.-H., Chou I.-C., Teng Y.-J. (2016). Piezoelectric tactile sensor for submucosal tumor detection in endoscopy. Sens. Actuators A Phys..

[4] Sharma S., Aguilera R., Rao J., Gimzewski J.K. (2019). Piezoelectric needle sensor reveals mechanical heterogeneity in human thyroid tissue lesions. Sci. Rep..

[5] Yip M.C., Yuen S.G., Howe R.D. (2010). A Robust Uniaxial Force Sensor for Minimally Invasive Surgery. IEEE Trans. Biomed. Eng..

[6] Noh Y., Liu H., Sareh S., Chathuranga D.S., Wurdemann H., Rhode K., Althoefer K. (2016). Image-Based Optical Miniaturized Three-Axis Force Sensor for Cardiac Catheterization. IEEE Sens. J..

[7] Lv C., Wang S., Shi C. (2019). A High-Precision and Miniature Fiber Bragg Grating-Based Force Sensor for Tissue Palpation During Minimally Invasive Surgery. Ann. Biomed. Eng..

[8] Li T., Pan A., Ren H. Reaction Force Mapping by 3-Axis Tactile Sensing with Arbitrary Angles for Tissue Hard-inclusion Localization. IEEE Trans. Biomed. Eng..

[9] Nagatom T., Miki N. A Flexible Tactile Sensor to Detect Stiffness Distribution without Measuring Displacement. Proceedings of the 2019 20th International Conference on Solid-State Sensors, Actuators and Microsystems & Eurosensors XXXIII (TRANSDUCERS & EUROSENSORS XXXIII).

[10] Aviles A.I., Alsaleh S.M., Hahn J.K., Casals A. (2017). Towards Retrieving Force Feedback in Robotic-Assisted Surgery: A Supervised Neuro-Recurrent-Vision Approach. IEEE Trans. Haptics.

[11] Ju F., Zhang Y., Yun Y., Guo H., Wei X., Zhu C., Zhang X., Bai D., Chen B. A Piezoelectric Tactile Sensor and Human-inspired Tactile Exploration Strategy for Lump Palpation in Tele-operative Robotic Minimally Invasive Surgery. Proceedings of the 2019 IEEE International Conference on Robotics and Biomimetics (ROBIO).

[12] Uribe D.O., Schoukens J., Stroop R. (2018). Improved tactile resonance sensor for robotic assisted surgery. Mech. Syst. Signal Process..

[13] Chalasani P., Wang L., Yasin R., Simaan N., Taylor R.H. (2018). Preliminary Evaluation of an Online Estimation Method for Organ Geometry and Tissue Stiffness. IEEE Robot. Autom. Lett..

[14] Goldman R.E., Bajo A., Simaan N. (2012). Algorithms for autonomous exploration and estimation in compliant environments. Robotics.

[15] Li L., Yu B., Yang C., Vagdargi P., Srivatsan R.A., Choset H. Development of an inexpensive tri-axial force sensor for minimally invasive surgery. Proceedings of the 2017 IEEE/RSJ International Conference on Intelligent Robots and Systems (IROS).

[16] Guan L., Weng L., Zhang X., Wu Z., Li Q., Liu L. (2020). Microstructures, electrical behavior and energy storage properties of Ag@shell/PVDF-based polymers: Different effects between an organic polydopamine shell and inorganic zinc oxide shell. J. Mater. Sci..

[17] Lukasz K.S. (2016). Concept, Implementation and Analysis of the Piezoelectric Resonant Sensor/Actuator for Measuring the Aging Process of Human Skin. Ph.D Thesis.

[18] Omata S., Murayama Y., Constantinou C.E. (2004). Real time robotic tactile sensor system for the determination of the physical properties of biomaterials. Sens. Actuators A Phys..

[19] Omata Y.S., Terunuma Y. Development of new type tactile sensor for detecting hardness and/or softness of an object like the human hand. Proceedings of the TRANSDUCERS’91: 1991 International Conference on Solid-State Sensors and Actuators. Digest of Technical Papers.

[20] Tian J., Xie Z. (2008). Dynamic contact stiffness of vibrating rigid sphere contacting semi-infinite transversely isotropic viscoelastic solid. Acta Mech. Solida Sin..

[21] Niemz M.H. (2005). Laser-Tissue Tissue Interactions Fundamentals and Applications.

[22] Zhao X.Y. (2018). Effects of Stiffness on Biological Behavior of Tumor Cells. Medical Review..

[23] Li M. (2015). Experimental Study and Numerical Simulation on Grasping Behavior between Fenestrated Mis Grasper and Soft Tissue. Master’s Thesis.

